# Distribution, Morphological Characterization, and Resiniferatoxin-Susceptibility of Sensory Neurons That Innervate Rat Perirenal Adipose Tissue

**DOI:** 10.3389/fnana.2019.00029

**Published:** 2019-03-14

**Authors:** Bo-Xun Liu, Ming Qiu, Peng-Yu Zong, Xu-Guan Chen, Kun Zhao, Yong Li, Peng Li, Wei Sun, Xiang-Qing Kong

**Affiliations:** Department of Cardiology, the First Affiliated Hospital of Nanjing Medical University, Nanjing, China

**Keywords:** perirenal adipose tissue, neural tracing, dorsal root ganglia, TRPV1, resiniferatoxin

## Abstract

Perirenal adipose tissue (PrAT) is a visceral adipose tissue involved in the pathogenesis of obesity and cardiovascular diseases via neural pathways. However, the origins, morphological characterization, and resiniferatoxin (RTX)-susceptibility of sensory neurons that innervate rat PrAT are yet unclear. Using neural tracing, an injection of DiI (1,1′-dioctadecyl-3,3,3′,3′-tetramethylindocarbocyanine perchlorate) into PrAT revealed that sensory neurons that innervate PrAT reside in T9-L3 dorsal root ganglia (DRG). Peak labeling occurred in T13 and L1 DRGs. Two distinct peaks were observed in cross-sectional areas of the labeled soma, and the mean cross-sectional area was 717.1 ± 27.7 μm2. Immunofluorescence staining for transient receptor potential cation channel subfamily V member 1 (TRPV1) separated DiI-positive neurons into three subpopulations: small TRPV1-negative, small TRPV1-positive, and large TRPV1-negative. Furthermore, the injection of RTX into PrAT reduced labeled cells by 36.7% where TRPV1-positive cells were the main target of RTX denervation. These novel findings provide a structural basis for future TRPV1-dependent and TRPV1-independent studies on the sensory innervation of PrAT, which may be of interest for future therapeutic obesity treatment and intervention.

## Introduction

Obesity has become a global epidemic and has contributed to the increasing prevalence of cardiovascular diseases (CVD) (Hu et al., 2001; Coutinho et al., 2011; Foster et al., 2011). Visceral obesity, characterized by an excessive accumulation of visceral adipose tissue, increases risks of hypertension and coronary heart disease (Després et al., 2008). Among various mechanisms, neural regulation plays an important role in the pathogenesis of these cardiovascular disorders. Adipose afferent reflex (AAR), which is marked by the overactivation of sensory neurons that innervate adipose tissue and by the subsequent activation of the sympathetic nervous system, contributes to the development of diet-induced obesity-associated hypertension (Xiong et al., 2012). Perirenal adipose tissue (PrAT), a fat pad located in the retroperitoneal space (Standring, 2005), has been recognized as a visceral adipose tissue that mediates CVD pathogenesis (Li et al., 2016; Ma et al., 2016; Zhao et al., 2016). However, the origins and characterization of the sensory innervation of PrAT remain unknown. Somas of sensory neurons that innervate tissues can be observed in dorsal root ganglia (DRG) using fluorescent neural tracers such as DiI (1,1′-dioctadecyl-3,3,3′,3′-tetramethylindocarbocyanine perchlorate) (Makarenko, 2014). Previous neural tracing studies have located sensory neurons projected into retroperitoneal viscera, such as the ureter and colon, in both thoracolumbar and sacral DRGs indicating dual sensory innervation (Robinson et al., 2004; Herweijer et al., 2014). We question whether dual sensory innervation also exists in PrAT.

Numerous neural somas reside in a single DRG, and morphological characterization has been used to distinguish between different sensory neurons. Based on the size of the soma, a sensory neuron can be classified as a large or small neuron (Sun et al., 2009). In addition to their different morphologies, large and small neurons have different cell surface receptors and different functions (Albisetti et al., 2017). Understanding the differences in neural cell morphologies facilitates functional studies of neurons. Thus, it is necessary to analyze the sizes of somas of sensory neurons that innervate PrAT to investigate disorders associated with PrAT.

The ablation of sensory neurons, also known as deafferentation, has been used in functional studies of sensory innervation, as it blocks the transmission of sensory signals from peripheral tissues to the spinal cord and brain. Among the numerous deafferentation techniques available, chemical denervation with capsaicinoids has been an accepted method in neurophysiological studies (Bartness et al., 2014). Capsaicinoids were originally discovered as bioactive compounds in chili peppers (Suzuki and Iwai, 1984). Among all of the known capsaicinoids, resiniferatoxin (RTX) is the most potent agonist of transient receptor potential cation channel subfamily V member 1 (TRPV1), a specific capsaicinoid receptor (Hergenhahn et al., 1975; Herweijer et al., 2014). Local injection with an overdose of RTX into peripheral tissues selectively destroys afferent nerves by activating TRPV1 (Herweijer et al., 2014). Although studies have used RTX to analyze the characteristics and functions of afferent nerves in peripheral tissues, studies on RTX denervation in adipose tissue are scarce.

In this study, we aimed to characterize primary afferent neurons that innervate rat PrAT by identifying the locations and morphologies of these neurons and by evaluating the expression of TRPV1 in neurons following intra-adipose administration of an overdose of RTX.

## Materials and Methods

### Animals

Adult male Sprague-Dawley (SD) rats weighing 320–350 grams (Vital River Biological, Beijing, China) were injected with DiI (Sigma-Aldrich Chemical Co., Unites States) and/or RTX (Sigma-Aldrich Chemical Co., United States). The rats resided in a temperature-controlled room on a 12-12 h light-dark cycle with standard chow and tap water provided *ad libitum*. All experiments were approved (Approval of Animal Ethical and Welfare Number IACUC-1712026) by the Experimental Animal Care and Use Committee of Nanjing Medical University (China) and were conducted in accordance with the Guide for the Care and Use of Laboratory Animals (publication 85–23, revised 1996; National Institutes of Health, Bethesda, MD, United States).

### DiI-Labeling of Sensory Neurons in PrAT

Six adult male SD rats were anesthetized via the inhalation of 3% isofluorane, and successful anesthetization was determined by a failure to evoke a tail or hindlimb reflex. When there was no response to a hindlimb or tail pinch, an incision was made on the abdominal midline to expose the abdominal cavity. Viscera that obstructed the perirenal fat were reflected to expose the retroperitoneal space. DiI was diluted to 1 mmol/L with dimethyl sulfoxide. Approximately 100 μl of DiI was injected into PrAT bilaterally using a 25-gage needle (5.0 μl per site, 10 sites for each side, two sides). Fat pads were inspected for any leakage into the surrounding internal organs by both visual inspection and stereo fluorescence microscopy. Fat pads were then rinsed with sterile 0.9% saline. Viscera were then repositioned back to their original positions. Abdominal muscles and skin were closed with sutures.

### Chemical Denervation of PrAT

Resiniferatoxin injections were administered 7 days after DiI tracing. Before deafferentation, RTX was dissolved in absolute ethanol to form a stock solution. The working solution of RTX, which contained 1% stock solution, 1% tween 80, and 98% normal saline, was injected into the PrAT. Twelve adult male SD rats were randomly assigned to one of two groups, the control group or the RTX group. The rats were anesthetized through the inhalation of 3% isoflurane. When a tail or hindlimb reflex was not evoked following a tail or hindlimb pinch, an incision was made on the abdominal midline to expose the abdominal cavity. Viscera that obstructed PrAT were reflected to expose the retroperitoneal space. Approximately 40 μl of RTX or vehicle solution was injected into PrAT bilaterally using a 25-gage needle (2.0 μl per site, 10 sites for each side, two sides). Fat pads were inspected for leakages into the surrounding internal organs and were rinsed with sterile 0.9% saline. Viscera were then repositioned back to their original positions. Abdominal muscles and skin were closed with sutures.

### Tissue Dissection and Preparation

The rats were under constant observation before being sacrificed 7 days after DiI injection or 3 days after RTX injection. Rats were deeply anesthetized via the inhalation of 5% isoflurane and were then perfused transcardially with 0.01 mol/L phosphate-buffered saline (PBS, 37°C). PrAT was removed along with the kidneys and paranephric adipose tissue, as PrAT is adjacant to these two tissues. Here, paranephric adipose tissue, also known as pararenal fat, is referred to as a retroperitoneal fat pad that is located in the posterior pararenal space. It lies posteriorly and posterolaterally to each kidney, and is superficial to Gerota’s fascia (Raptopoulos et al., 1997; Standring, 2005). These tissues were then fixed in 4% paraformaldehyde. The other internal organs were once again inspected for any leakages of DiI dye, and it was found that DiI was localized to the perirenal fat pads (Supplementary Figure S1A). The rats were then perfused transcardially with 4% paraformaldehyde (4°C). DRGs at spinal levels of T9-S2 were removed and as much excessive connective tissue and epineurium as possible were removed before post-fixing the DRGs in 4% paraformaldehyde. Subsequently, DRGs were removed and post-fixed in paraformaldehyde overnight at 4°C and then dehydrated in 20 and 30% sucrose at 4°C for 24 h.

For cell counting, 20 μm longitudinal DRG sections were cut on a cryostat and directly mounted onto three slides with every fourth section on the same slide. This procedure yielded approximately 30 sections with each slide containing 10 sections. For immunofluorescence staining, 8 μm longitudinal sections were mounted directly onto slides. For kidney morphology after RTX injection, 4 μm longitudinal sections were mounted directly onto slides and stained with Periodic Acid-Schiff (PAS) stain.

### Immunofluorescence Staining

The tissue slides were processed for immunofluorescence staining. The tissue slides were rinsed with PBS and blocked for 1 h with 2.5% bovine serum albumin (BSA). For the DRGs, the slides were incubated with rabbit primary anti-TRPV1 antibody (Alomone Lab, Israel) for 24 h at 4°C overnight. For the adipose tissue, the slides were incubated with pan-axonal antibodies (SMI-312, BioLegend, San Diego CA, United States) and sheep primary anti-tyrosine hydroxylase (TH) antibody (Pel-Freez Biologicals, Rogers, AR, United States) for 24 h. After incubation with the primary antibody, the tissue slides were rinsed with PBS and incubated with Alexa Flour 488-conjugated anti-rabbit secondary antibody (Jackson Immunoresearch, West Grove, PA, United States; at a dilution of 1:200), FITC-conjugated anti-sheep secondary antibody (Jackson Immunoresearch; at a dilution of 1:200), Alexa Flour 594-conjugated anti-mouse secondary antibody (Invitrogen, Waltham, MA, United States; at a dilution of 1:200), or Cy3-conjugated anti-mouse species secondary antibody (Jackson Immunoresearch; at a dilution of 1:200) for 1 h. Then, the tissue slides were rinsed with PBS and incubated in FluoroGold with DAPI (Invitrogen) and images were captured.

To mark the cytoskeleton within the renal cortex, renal tissue slides were incubated with FITC-conjugated phalloidin (Servicebio Co., Ltd., China). After incubation with phalloidin for 1 h, the tissue slides were rinsed with PBS and incubated in FluoroGold with DAPI, and images were captured.

### Quantitative Analysis of Immunofluorescent Signals

Images were viewed and captured at a magnification of 100x or 200x with a Zeiss Axio Imager A2 microscope (Zeiss, Germany) with FITC (for TRPV1), Rhodamine (for DiI), and/or DAPI fluorescence filters. Photographs of the DRGs were taken at 50–150 ms using Zen Software 2 (Zeiss, Germany). The images were evaluated and overlaid using Zen Software 2 and ImageJ software ver.1.51j8 (National Institutes of Health, Bethesda, MD, United States). To quantify DiI-labeling, every fourth section was captured at 100x magnification, and somas with red fluorescence were marked using the “multi-point” tool in ImageJ to avoid counting the same neuron more than once. The estimated total number of DiI-positive neurons per ganglion was calculated using the following formula: total number of neurons = average number of neurons per section × number of sections × 3.

Distributions of absolute numbers, sizes, and receptors of neurons were averaged across each ganglion for all of the animals. To quantify TRPV1 immunofluorescent signals, sections were acquired at 200 × magnification with a fluorescence microscope and the integrated optical density (OD) in each soma was measured with ImageJ software ver.1.51j8. The results were expressed in arbitrary units as previously described by Lalancette-Hébert et al. (2009).

### Physiological Assessment of Adipose Tissue

An *ex vivo* assay of basal lipolysis rates was conducted based on an article by Schreiber et al. (2017). Seven days after RTX or vehicle injection, fresh PrAT was dissected from the rats and placed into phenol red-free DMEM (Gibco, Waltham, MA, United States) supplemented with 2% fatty acid (FA)-free BSA. PrAT was cut into small pieces and transferred into DMEM supplemented with 2% FA-free BSA in 96-well plates for 30 min (preincubation). To analyze basal lipolysis, the adipose tissue was transferred into 150 μl fresh media and incubated for an additional 60 min. Then, glycerol content in the media was analyzed using a glycerol assay kit (Nanjing Jiancheng Bioengineering Institute, China). Data are expressed as the level of glycerol per *g* of tissue mass.

The measurement of AAR was based on an article by Xiong et al. (2012). In brief, the left renal sympathetic nerve was isolated, cut distally to abolish its afferent activity, placed on silver electrodes attached to an AC/DC differential amplifier (Warner Instruments, Hamden, CT, United States) and immersed in mineral oil. The RSNA was amplified, filtered with a band-pass of between 60 and 3000 Hz, recorded with a PowerLab 8/35 System (ADInstruments, Australia) and stored on a hard disk until analysis. After recording the baseline RSNA level, capsaicin (1.0 nmol/μl) was injected into both PrAT at a rate of 4.0 ul/min for 2 min at 4 sites. Nerve activity was expressed as the percent change in integrated RSNA values from the baseline.

### Statistical Analysis

An unpaired *t*-test or Mann-Whitney U-test was used to compare numeric values of the two groups. A Chi-squared test was used to compare rates of the two groups. A one-way analysis of variance was used to draw multiple comparisons followed by a *post hoc* Bonferroni test. A correlation analysis was performed with a Pearson Correlation Coefficient test. All data are expressed as the means ± standard error (SE). We considered *p*-values of < 0.05 to be statistically significant.

## Results

### Distribution of DiI-Labeled Sensory Neurons in Spinal DRGs

**FIGURE 1:**
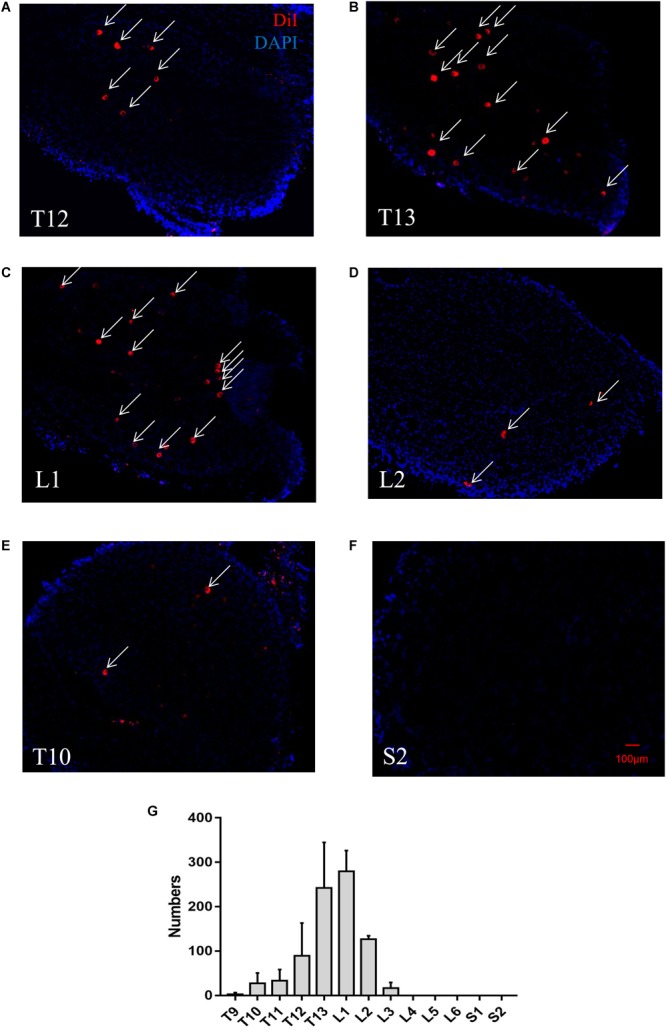
A representative retrograde trace of a DRG after DiI injection into perirenal adipose tissue (PrAT) *in vivo*. **(A–D)** Numerous DiI-labeled neurons (indicated by arrows) were found in the T12-L2 DRGs. **(E)** Few DiI-labeled neurons were found in the T10 DRG. **(F)** No DiI-positive neurons were found in the sacral DRG. **(G)** The numbers of PrAT-innervating neurons in DRGs throughout the spine. The largest numbers of neurons were found in the T13 and L1 DRGs.

Our immediate inspection of retroperitoneal space using a stereo-fluorescence microscopy shows that DiI was restrained to the PrAT without leaking into the retroperitoneal space or into other internal organs (Supplementary Figure S1B). Seven days after bilateral intra-adipose injections of DiI were made (Supplementary Figure S1C), DiI-labeled cell bodies were visualized as red punctate fluorescence in DRG slices from six rats. DiI-labeled pseudounipolar neurons were distributed unevenly in DRG at different spinal levels. DRGs in T12-L2 contained the most DiI-labeled neurons (Figure 1A–D); DRGs in T9, T10, L3 contained fewer DiI-labeled neurons (Figure 1E); and DRGs in L4-S2 contained no DiI-labeled neurons (Figure 1F). This DiI-labeling pattern of DRGs in various spinal segments was consistent across the six rats.

In addition, local injections of DiI into the kidneys only marked cells in the renal cortex around the injection point (Supplementary Figures S2A,B). Sensory neurons projecting to the kidneys failed to be labeled by the fluorescent tracer (Supplementary Figures S2C–H). These findings indicate that DiI-labeled cell somas are afferent neurons that specifically innervate PrAT.

To quantify the distribution of PrAT-innervating neurons in DRGs in various spinal segments, the number of DiI-labeled neurons in all of the DRG slices was counted. The number of DiI-labeled neurons was highest in the T13 and L1 DRGs with 242.6 ± 45.8 and 279.8 ± 23.4 DiI-positive neurons, respectively, whereas the number of DiI-labeled neurons in the T9–T12, L2, and L3 DRGs was measured at less than 150. There were no DiI-labeled neurons in the L4–S2 DRGs (Figure 1G).

### DiI-Labeled Sensory Neurons Can Be Classified by Cell Size

**FIGURE 2:**
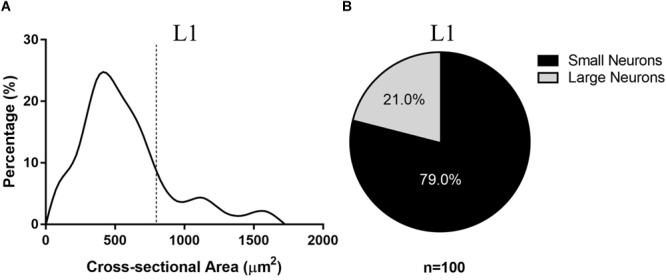
Cross-sectional areas of DiI-labeled cells in rat DRGs. **(A)** The cross-sectional areas of 100 labeled neurons, which were randomly selected from the L1 DRGs of six rats, are plotted against the number of cells observed in each 50 μm^2^ bin to determine the distribution of cross-sectional areas of the neurons. **(B)** Of the 100 labeled neurons, 79% were between 100 and 800 μm^2^.

Because the L1 DRG was found to be predominant in PrAT, the L1 DRG was used for a cross-sectional analysis of somas of DiI-labeled neurons (Figure 2A). Cross-sectional areas of DiI-labeled somas ranged from 90 to 1800 μm^2^ with a mean of 717.1 ± 27.7 μm^2^ (*n* = 100). A histogram of the cross-sectional areas reveals two distinct peaks at 300–450 μm^2^ and 1050–1200 μm^2^, and the nadir between the two peaks was recorded at 975 μm^2^. In addition, 21.0% of the neurons had a cross-sectional area of > 800 μm^2^ and 79.0% of the neurons had a cross-sectional area of < 800 μm^2^ (Figure 2B).

### TRPV1-Positive Neurons Are Small Neurons Among PrAT-Innervating Neurons

**FIGURE 3:**
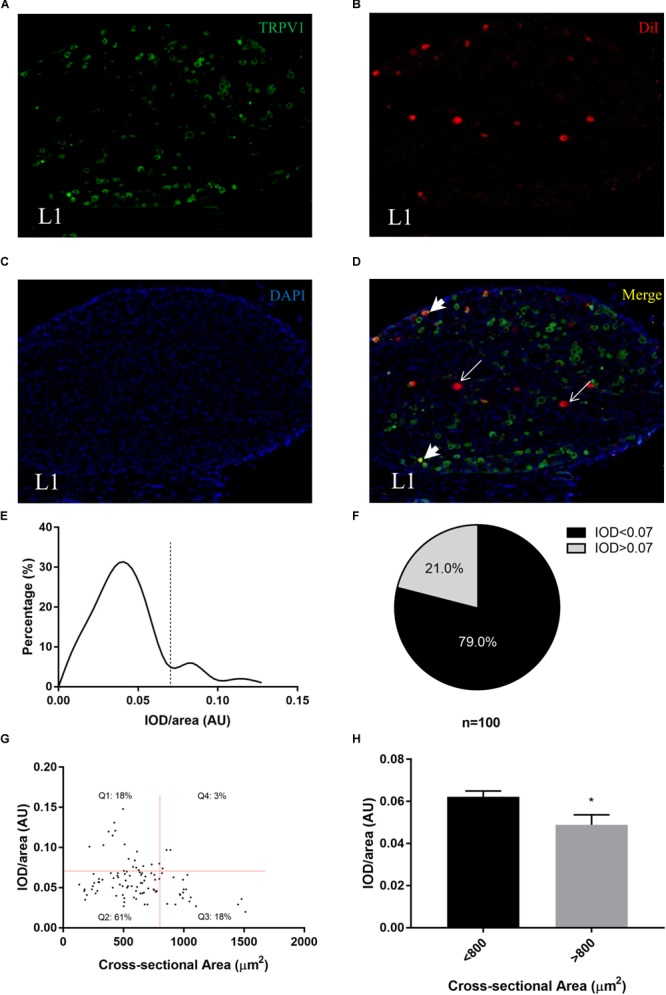
Transient receptor potential cation channel subfamily V member 1 (TRPV1) expression levels in PrAT-innervating sensory neurons. **(A–D)** Immunofluorescence images show the differential expression of TRPV1 among DiI-positive neurons. **(E,F)** The histogram shows that TRPV1-positive cells were a minority among the cells. **(G)** The scatter plot shows that TRPV1-positive cells are mainly small neurons. **(H)** The intensity of immunofluorescence was lower for small neurons than for large neurons.

Next, the expression of TRPV1 in each neuronal cell of the L1 DRG was analyzed by immunofluorescence. TRPV1 expression was detected as green fluorescence and was detected in the L1 DRG (Figure 3A–D). Interestingly, the intensity of green fluorescence among the DiI-labeled cells varied. Average ODs of the DiI-labeled neurons ranged from 0.02 to 0.15 arbitrary units. A histogram of average ODs of the DiI-labeled neurons shows a peak at 0.0375 arbitrary units (Figure 3E). In addition, 21% of the DiI-labeled neurons had average ODs of > 0.07 (Figure 3F).

To further characterize the TRPV1-positive cells, we created a scatter plot with the cross-sectional area of a neuron as the independent variable and the average OD as the dependent variable. We found that neural somas with high average ODs were more likely to be small rather than large (Figure 3G), and thus we separated the DiI-positive neurons into three subpopulations: small TRPV1-negative, small TRPV1-positive, and large TRPV1-negative. Furthermore, the mean of the average ODs of the small neurons was higher than the mean of the average ODs of the large neurons (*p* < 0.05, Figure 3H).

### Local Injection of RTX Destroyed the Afferent Innervation of PrAT

**FIGURE 4:**
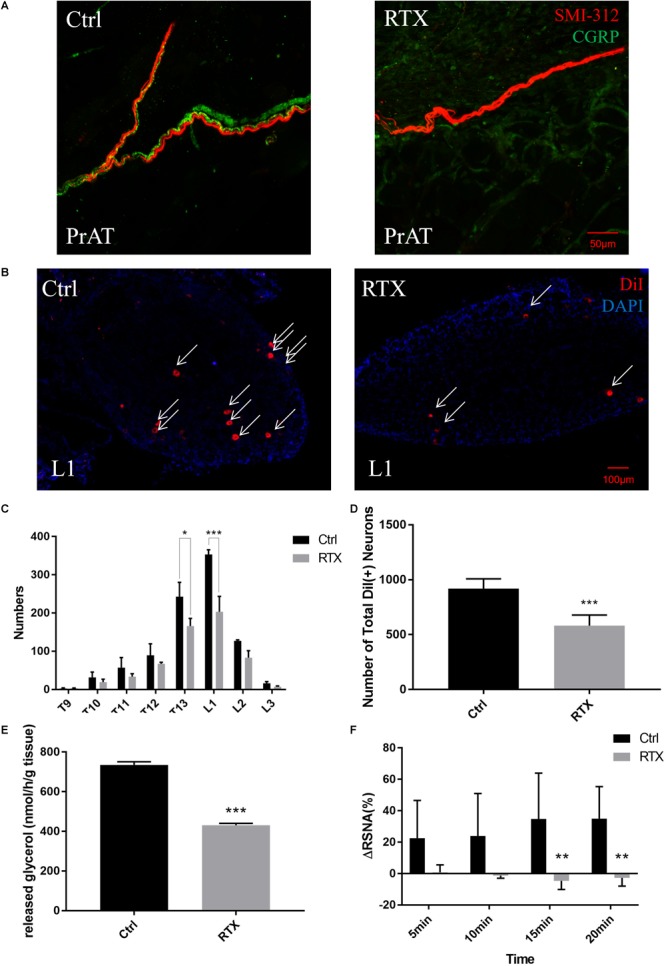
Local injection of resiniferatoxin (RTX) into the structure of afferent nerves within PrAT and the functions of PrAT. **(A)** The density of afferent nerves within PrAT was determined by immunostaining CGRP and SMI-312. **(B–D)** The number of neurons in the T9-L3 DRGs reduced after injection with RTX. **(E)** The lipolysis rate measured by the glycerol release of PrAT was attenuated after deafferentation with RTX. **(F)** Local injections of capsaicin into PrAT failed to evoke adipose afferent reflex (AAR) in the RTX group.

Seven days after the local injection of RTX into PrAT, the density of CGRP-positive afferent nerve axons in PrAT was reduced relative to the control group (Figure 4A). Efferent nerve axons were intact following the RTX injection (Supplementary Figure S3). Moreover, no obvious deafferentation was detected in either the kidneys (Supplementary Figure S4) or paranephric adipose tissue (Supplementary Figure S5).

Despite the injection of RTX into PrAT 7 days after DiI tracing, red punctate fluorescence remained in the DRG cell bodies (Figure 4B). However, rats in the RTX group had fewer DiI-labeled neurons in the T9-L3 DRGs than rats in the control group (Figure 4C and Table 1). Among these DRGs, the decrease in DiI-labeled cells observed was significant for T13 and L1 DRG. The total number of DiI-labeled cells in the RTX group significantly decreased by 36.7% relative to the control group (*p* < 0.001, Figure 4D and Table 1).

**Table 1:** The number of DiI-labeled perirenal-innervating neurons in T9-L3 DRGs of the RTX group and control group.

	Control	RTX	Reduction	*P*-value
T9	2.5 ± 3.99	3 ± 1.9	/	0.999
T10	31.5 ± 34.3	19 ± 19.5	39.7%	0.999
T11	57 ± 64.82	33.5 ± 18.82	41.2%	0.999
T12	89.5 ± 73.54	67 ± 9.8	25.1%	0.999
T13	242.67 ± 91.54	165.83 ± 48.91	31.7%	0.047
L1	353 ± 30.69	203 ± 99.2	42.5%	<0.001
L2	127 ± 6.66	83.03 ± 45.12	34.6%	0.876
L3	16.33 ± 10.09	7 ± 5.25	57.1%	0.999
Sum	919.5 ± 35.98	581.4 ± 39.43	36.7%	<0.001

*Reductions of neurons in T9 DRG were omitted because the number of labeled neurons in RTX was higher than that of the control group.*

To evaluate the functional impact of local RTX injection on PrAT, lipolysis rates and AAR were assessed. The PrAT of the RTX group presented a lower rate of glycerol release *in vitro* than that of the control group (*p* < 0.001, Figure 4E). Furthermore, the local injection of capsaicin elevated renal sympathetic nerve activity (RSNA) in normal rats. The same reflex failed to be evoked by the injection of capsaicin into rats of the RTX group. The difference in increased RSNA levels was significant 15 and 20 min after the injection of capsaicin (*p* < 0.01, Figure 4F).

### TPRV1-Positive Neurons Are the Main Targets of RTX Denervation

**FIGURE 5:**
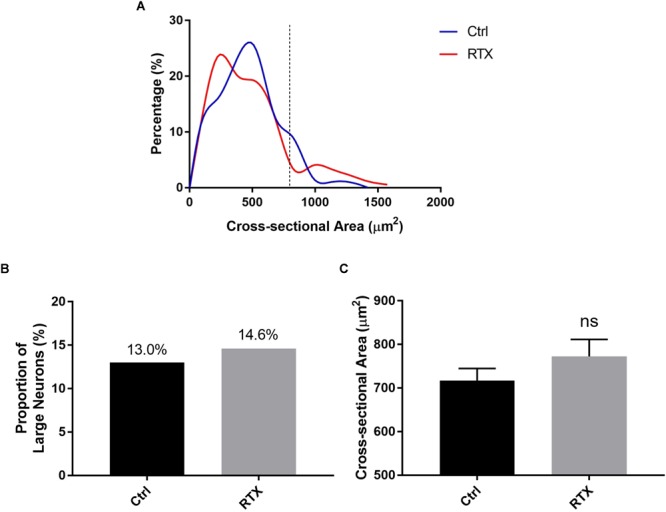
Alterations in PrAT-innervating sensory neurons following denervation with RTX. **(A)** Histogram of cross-sectional areas of somas in DRGs after injection with RTX. **(B,C)** There was a decrease in the proportion of small neurons and an increase in the mean cross-sectional area of somas in DRGs after injection with RTX.

In addition to the decreased number of DiI-positive neurons observed, the shape of the histogram of cross-sectional areas of the RTX group is different from that of the control group (Figure 5A). However, the proportion of small cells was only slightly lower in the RTX group (*p* = 0.66, Figure 5B). Similarly, the average cross-sectional area of DiI-labeled neural somas was slightly higher for the RTX group than for the control group (*p* = 0.38, Figure 5C).

**FIGURE 6:**
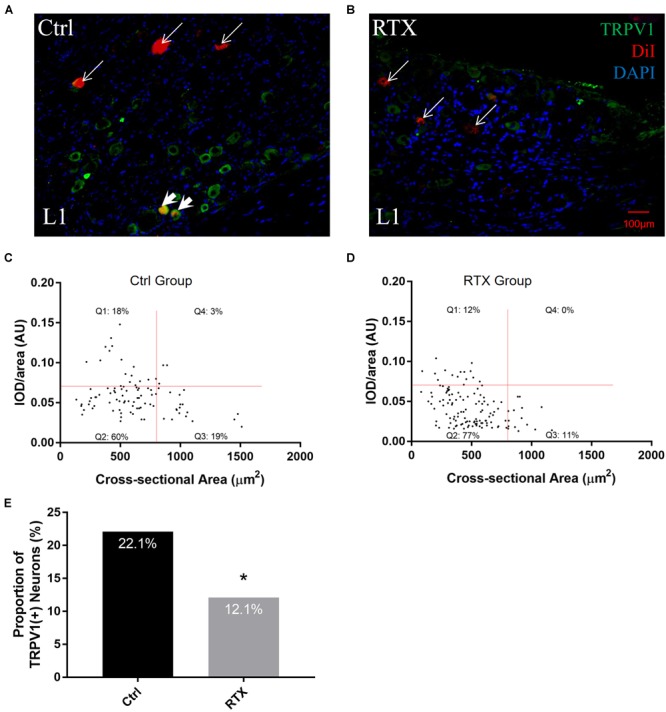
Alterations in TRPV1 expression levels of PrAT-innervating sensory neurons following denervation with RTX. **(A,B)** Fluorescence images show a decrease in the number of TRPV1- and DiI-positive cells in the RTX group compared to the control group. **(C–E)** The scatter plot shows that the percentage of TRPV1-positive cells was reduced among DiI-positive cells.

To measure changes in DiI-positive neurons in the RTX group, an immunofluorescence staining of TRPV1 was performed, showing that there were fewer small TRPV1-positive neurons in the RTX group than in the control group (Figure 6A,B). To characterize the alteration of TRPV1-positive cells, we again created a scatterplot with the cross-sectional area of a neuron as the independent variable and the average OD as the dependent variable. The portion of TRPV1-positive cells was significantly lower for the RTX group (12%) than for the control group (22%) (*p* < 0.05, Figure 6C–E).

## Discussion

In this study, we determined the locations of DRGs that innervate rat PrAT and we characterized the morphologies of sensory neuronal somas in these DRGs. We also demonstrated the relationship between the size of a neuronal soma and the level of TRPV1 expression, and we determined the extent and main target of RTX denervation in PrAT.

Although PrAT is a fat pad in retroperitoneal space, DiI tracing results show that sensory neurons that innervate rat PrAT reside in thoracolumbar DRGs only. Notably, this differs from dual innervation patterns found in most internal organs, including the uterus, colon, and other urogenital organs (Marfurt and Echtenkamp, 1991; Robinson et al., 2004; Ivanusic et al., 2013; Herweijer et al., 2014). For these organs, dual afferent innervation is linked to dual autonomic efferent innervation. Sensory nerve fibers that accompany sympathetic nerve fibers originate from thoracolumbar DRGs whereas sensory nerve fibers that accompany para-sympathetic nerve fibers originate from sacral DRGs (Mei, 1983). However, Bartness et al. (2014) demonstrated that the efferent innervation of white adipose tissue is exclusively sympathetic and that there is a lack of para-sympathetic innervation of white adipose tissue (Giordano et al., 2006). PrAT is an adipose tissue composed of both white and brown adipose tissues, and the innervation of PrAT had not been determined. Our data suggest that the lack of sacral sensory innervation in PrAT may be due to a lack of para-sympathetic innervation, and this may explain the single peak distribution of sensory neurons found in this study.

To determine in which DRG PrAT-innervating sensory neurons reside, we estimated the number of PrAT-innervating sensory neurons in every DRG from T9–S4. We found DiI-labeled neurons in the T10–L3 DRGs, and DiI-label peaks were found in the T13 and L1 DRGs. This distribution is inconsistent with earlier retrograde tracing studies of pig PrAT, in which a neural tracer label peak was observed in L1–L3 paravertebral ganglia (Czaja et al., 2002). This inconsistency may be attributable to the following reasons. First, anatomical origins of efferent and afferent nerves differ. In pigs, nerve fibers in L1–L3 paravertebral ganglia constitute only a portion of the efferent nerve fiber network while other portions originate from celiac, superior mesenteric, inferior mesenteric, and aortorenal ganglia (Czaja et al., 2002). In rats, the sensory nerve fibers that accompany efferent nerve fibers originating in these sympathetic ganglia may not originate from L1–L3 DRGs. Second, this inconsistency may be due to differences in the spine structures of pigs and rats. There are 14–17 (an average of 15.42) thoracic and 4–8 lumbar (an average of 6.12) vertebrae in pigs (Rohrer et al., 2015) whereas rats only have 13 thoracic and six lumbar vertebrae. The number of DRGs also differs between pigs and rats, and thus a particular spinal DRG found in pigs is not necessarily equivalent to the same spinal DRG in rats.

Furthermore, because PrAT is positioned adjacent to the kidneys (Standring, 2005), the sensory innervation of PrAT should be compared to the sensory innervation of the kidneys. A retrograde tracing study of canine kidneys showed that renal afferent nerves originate in T5–L3 DRGs and a tracer label peak was observed in the T10–L2 DRGs (Li et al., 2005). The range of the tracer label peak found in this canine kidney study is similar to the range of the DiI-label peak found in our rat PrAT study. This similarity in neuron labeling may be due to the proximity of PrAT to the kidneys or due to sharing and/or crosstalk in the afferent neural pathway.

We analyzed the sizes of sensory nerve somas in DRGs that innervate PrAT, and we found that these neurons can be classified into two groups by soma size. Importantly, the majority of sensory neurons that innervated rat PrAT were small neurons of < 800 μm^2^. Most sensory information passed via the DRG is conveyed by small diameter, unmyelinated, slow-conducting C-fibers and large diameter, lightly myelinated Aδ fibers (Sun et al., 2009; Herweijer et al., 2014). A recent study revealed correlations between the sizes of somas and their specific neurochemistries (Tan et al., 2008). We analyzed TRPV1 expression in DiI-positive neurons, and we identified three subpopulations of sensory neurons that innervate PrAT: small TRPV1-negative, small TRPV1-positive, and large TRPV1-negative neurons. We also found that most of the TRPV1-positive neurons that innervate PrAT are small neurons. Although the small neurons in DRGs are initially identified as somas for C-fibers which encodes nociceptive signals (Sun et al., 2009), we discovered that these neurons are involved in regulation of lipolysis and sympathetic nerve activities using RTX deafferentation.

In this study, we also evaluated the effects of RTX denervation on sensory neurons in DRGs. Immunofluorescence staining showed that local injection of RTX remarkably decreased the density of CGRP-positive neural fibers within PrAT. Meanwhile, afferent neural fibers were not destroyed within the kidneys (adjacent tissue inside Gerota’s fascia) or paranephric adipose tissue (adjacent tissue outside Gerota’s fascia). These results demonstrate that RTX denervation was remarkable and specific. Hence, we also expected to find a dramatic reduction in the number of sensory neurons upon the injection of an overdose of RTX into PrAT. However, we found only a 36.7% reduction in DiI-labeled neurons, indicating that RTX destroyed only a small portion of the afferent nerve network in PrAT. This difference might be attributed to the composition of nerve fibers in PrAT. CGRP-positive neural fiber forms part of afferent nerve fibers. CGRP-positive and CGRP-negative afferent fibers may have different responses to RTX denervation. Meanwhile, RTX deafferentation led to a remarkable alteration of the functions of PrAT revealed as reduced lipolysis rates and as a capacity to evoke AAR following RTX injection. Our data illustrate the important role of RTX-responsive afferent nerves in PrAT physiology. On the other hand, our data support (Mei, 1983; Bartness et al., 2012) finding that destroying the afferent nerve decreases intra-adipose sympathetic nerve activity, inhibiting the lipolysis of adipose tissue.

Finally, our study highlights a potential relationship between TRPV1 and RTX denervation. Although both the mean cross-sectional area of DiI-labeled neurons and the proportion of small neurons only slightly decreased in the RTX group, a scatter plot analysis showed a decrease in the proportion of TRPV1-positive small neurons upon RTX injection. Because RTX is an ultrapotent ligand of TRPV1, the TRPV1 channel may mediate the chemical denervation of the fibers of small neurons. Interestingly, the number of RTX-responsive neuronal cells (36.7%) was greater than that of TRPV1-positive neurons (22.1%). This means that some TRPV1-negative neurons might also undergo capsaicinoid denervation, as RTX was shown to specifically bind to sensory neurons from TRPV1 null mice (Roberts et al., 2004). Nevertheless, TRPV1-positive neurons are the main targets of RTX denervation, as TRPV1-positive cells underwent the sharpest decline among all subpopulations of neuronal cells. In addition, our finding does not contradict the neurophysiological or functional effects of high concentrations of capsaicinoids on afferent nerves shown in previous studies (Shi and Bartness, 2005; Xiong et al., 2012; Nguyen et al., 2018). The functions observed in previous studies may have been mediated by small TRPV1-positive neurons. In addition, purinergic receptors (Tozzi and Novak, 2017) and Mas-related G protein-coupled receptors are expressed in TRPV-1-negative neurons (Bader et al., 2014), and thus TRPV1-negative neurons may have different functions than TRPV1-positive neurons. The properties of RTX-resistant, TRPV1-negative neurons require further investigation.

## Conclusion

**FIGURE 7:**
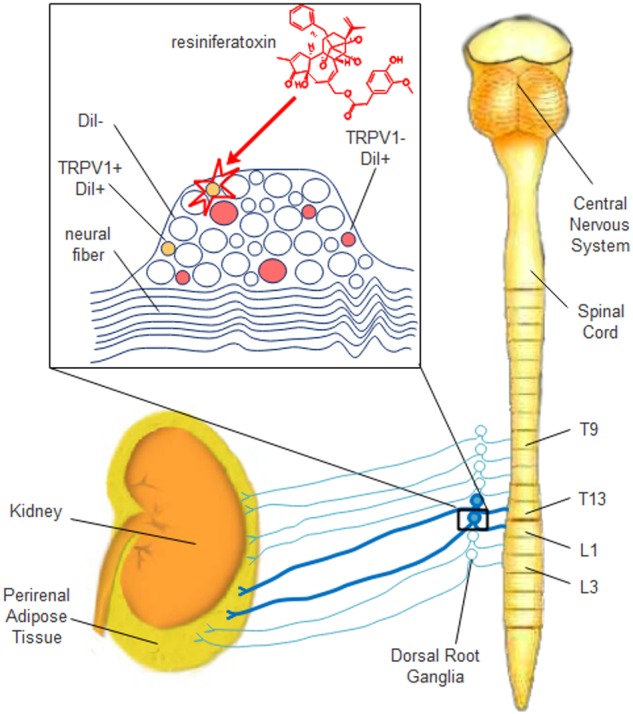
Schematic diagram of the distribution, morphological characterization, and resiniferatoxin-susceptibility of sensory neurons that innervate rat PrAT. In this study, sensory neurons that innervate rat PrAT were localized to the T9–L3 DRGs, and the majority of these neurons resided in the T13 and L1 DRGs. Injection with RTX into PrAT selectively destroyed TRPV1-positive small neurons.

In conclusion, this study identified the spinal DRGs involved in the primary afferent innervation of rat PrAT. We found the number of sensory neurons to peak in T13 and L1 DRGs. We also determined that of neuron subpopulations, small TRPV1-positive neurons are the main target of RTX denervation (Figure 7). These novel findings provide a structural basis for future TRPV1-dependent and TRPV1-independent studies on the primary afferent innervation of PrAT, which may be of interest for the development of future therapeutic obesity and CVD interventions.

## Data Availability

All datasets generated for this study are included in the manuscript and/or the Supplementary Files.

## Author Contributions

B-XL, WS, and X-QK designed the study and wrote the paper. B-XL, MQ, P-YZ, X-GC, KZ, and YL performed and analyzed the experiments. PL provided technical assistance and contributed to the preparation of figures. All authors analyzed the results and approved the final version of the manuscript.

## Conflict of Interest Statement

The authors declare that the research was conducted in the absence of any commercial or financial relationships that could be construed as a potential conflict of interest.

## Supplementary Material

FIGURE S1 Visual and microscopic inspection of DiI injection and leakages. **(A)** Sites of bilateral DiI injections into each perirenal fat pad were apparent via visual inspection. **(B)** DiI fluorescence was restrained to the perirenal fat pad upon correct injection. Fluorescence is clearly detected with an enlarged image. No band of pigmentation or “floating” punctate DiI fluorescence was detected. **(C)** In contrast, when there were leakages of the tracer, a band of pigmentation (thick arrows) and “floating” punctate DiI fluorescence (thin arrows) was discovered in the retroperitoneal space.

FIGURE S2 The local injection of DiI into the kidney failed to label DRG neurons. **(A,B)** The fluorescence of the tracer was detected around injection sites of the kidney. **(C–H)** No DiI-labeled neurons were detected in the T9-L3 DRGs.

FIGURE S3 Efferent nerve fibers remained intact after the RTX injection. The density of TH-positive efferent nerves after RTX treatment **(B)** did not alter in comparison with control group **(A)**.

FIGURE S4 Effects of the deafferentation of PrAT by RTX on the kidneys. Injury occurred within glomeruli (glomerulosclerosis marked by arrows) with the leakage of RTX **(C)**. In our study, RTX treatment **(A)** did not result in pathological changes in the kidneys **(B)**.

FIGURE S5 The deafferentation of PrAT using RTX **(B)** did not alter the density of sensory nerve fibers within paranephric adipose tissue compared to the control group **(A)**.
