# Supplementary figures and images for: Distribution, Morphological Characterization, and Resiniferatoxin-Susceptibility of Sensory Neurons That Innervate Rat Perirenal Adipose Tissue
Source: Front Neuroanat. 2019 Mar 14;13:29. doi: 10.3389/fnana.2019.00029 (PMC6427091; doi:10.3389/fnana.2019.00029)

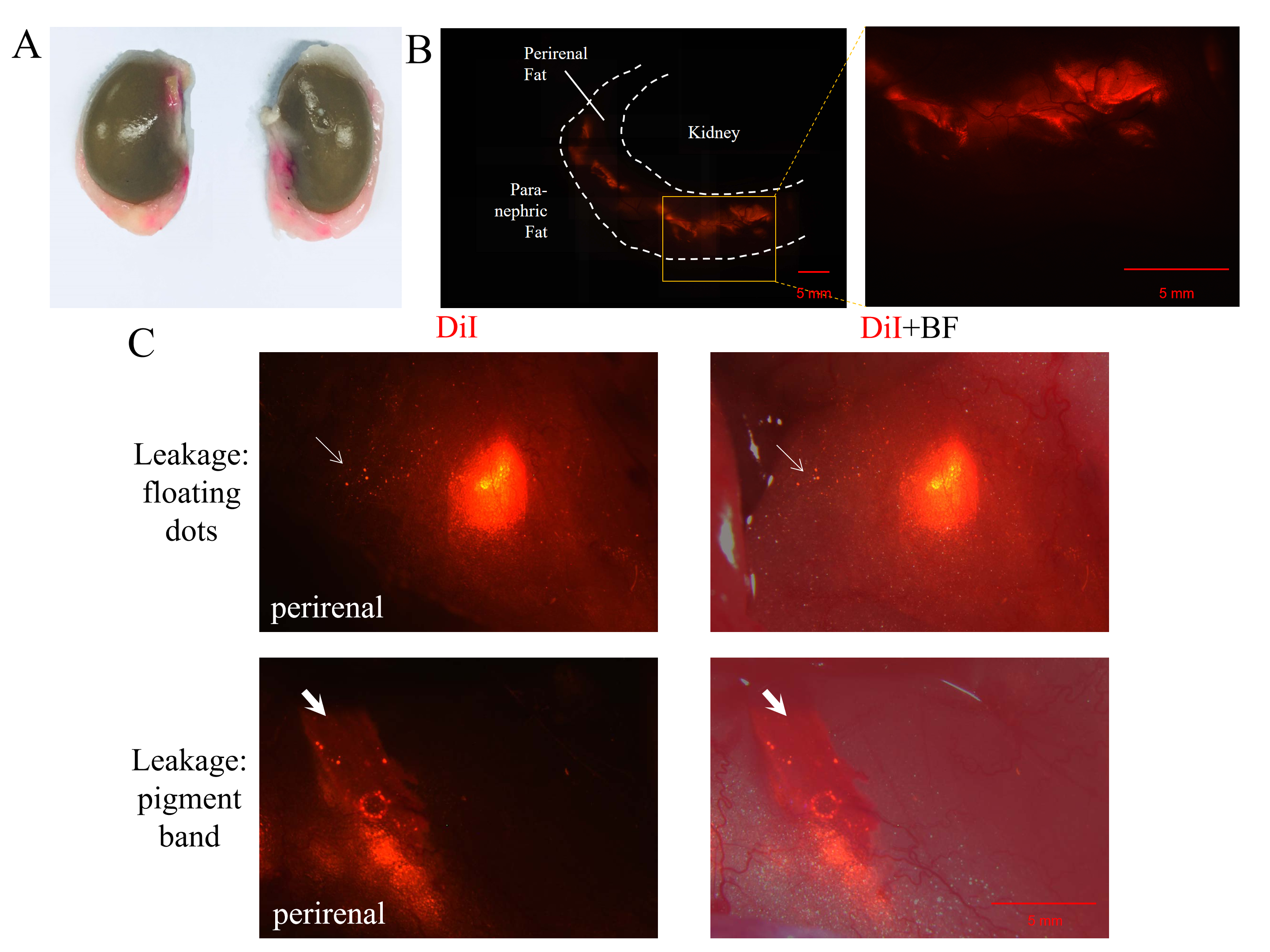

Supplement: FIGURE S1 — Visual and microscopic inspection of DiI injection and leakages. (A) Sites of bilateral DiI injections into each perirenal fat pad were apparent via visual inspection. (B) DiI fluorescence was restrained to the perirenal fat pad upon correct injection. Fluorescence is clearly detected with an enlarged image. No band of pigmentation or “floating” punctate DiI fluorescence was detected. (C) In contrast, when there were leakages of the tracer, a band of pigmentation (thick arrows) and “floating” punctate DiI fluorescence (thin arrows) was discovered in the retroperitoneal space. [file Image_1.TIF]

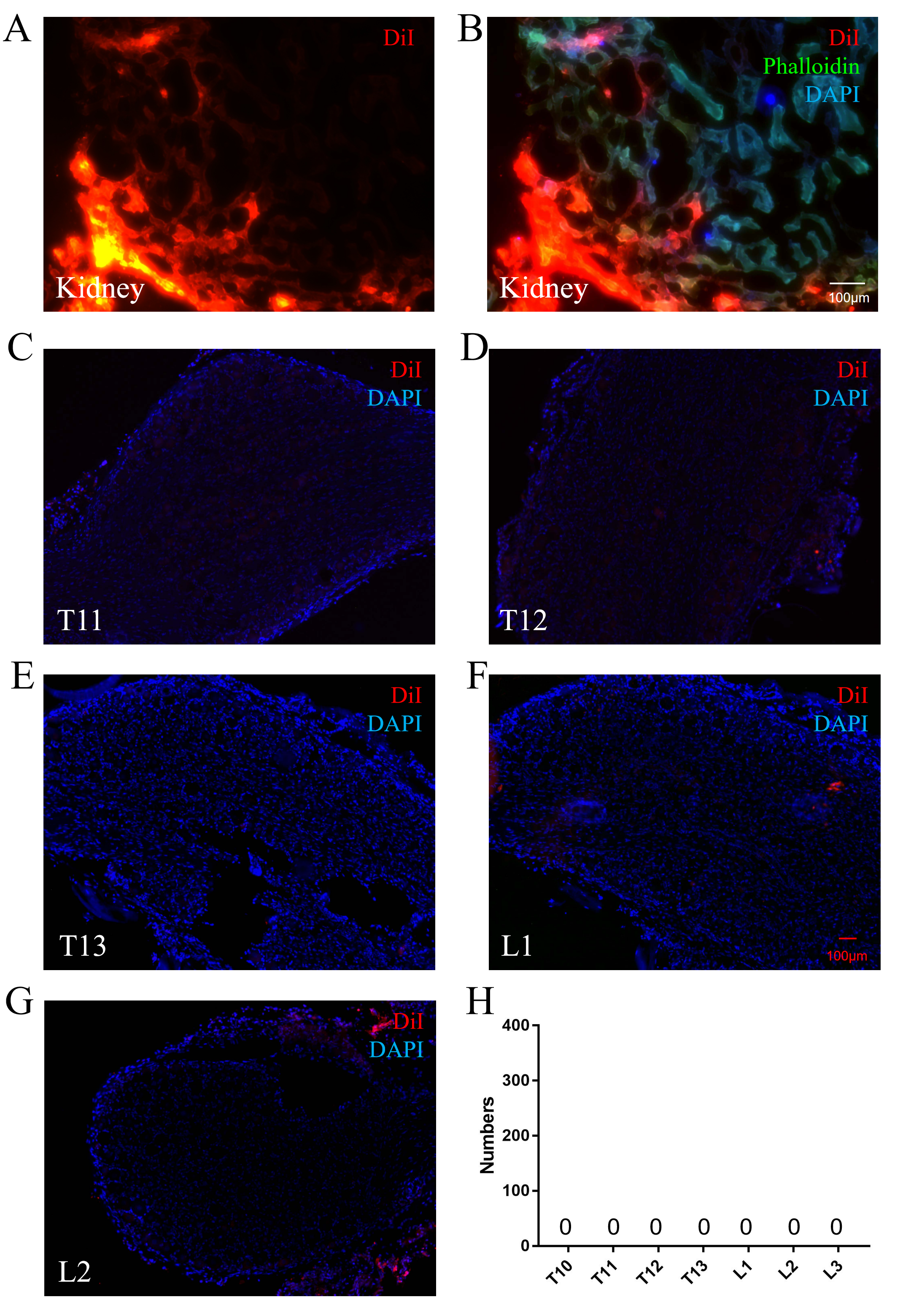

Supplement: FIGURE S2 — The local injection of DiI into the kidney failed to label DRG neurons. (A,B) The fluorescence of the tracer was detected around injection sites of the kidney. (C–H) No DiI-labeled neurons were detected in the T9-L3 DRGs. [file Image_2.TIF]

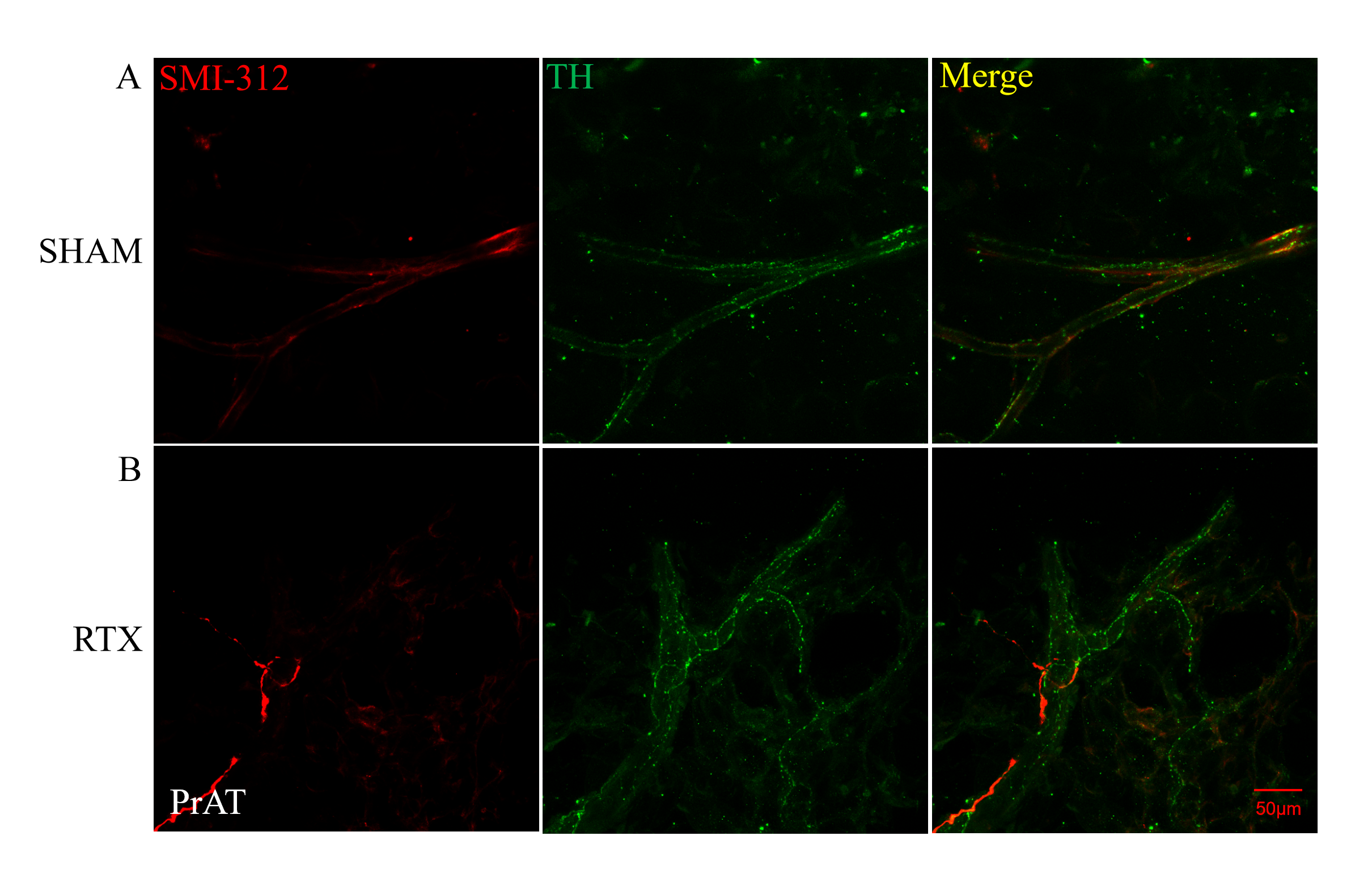

Supplement: FIGURE S3 — Efferent nerve fibers remained intact after the RTX injection. The density of TH-positive efferent nerves after RTX treatment (B) did not alter in comparison with control group (A). [file Image_3.tif]

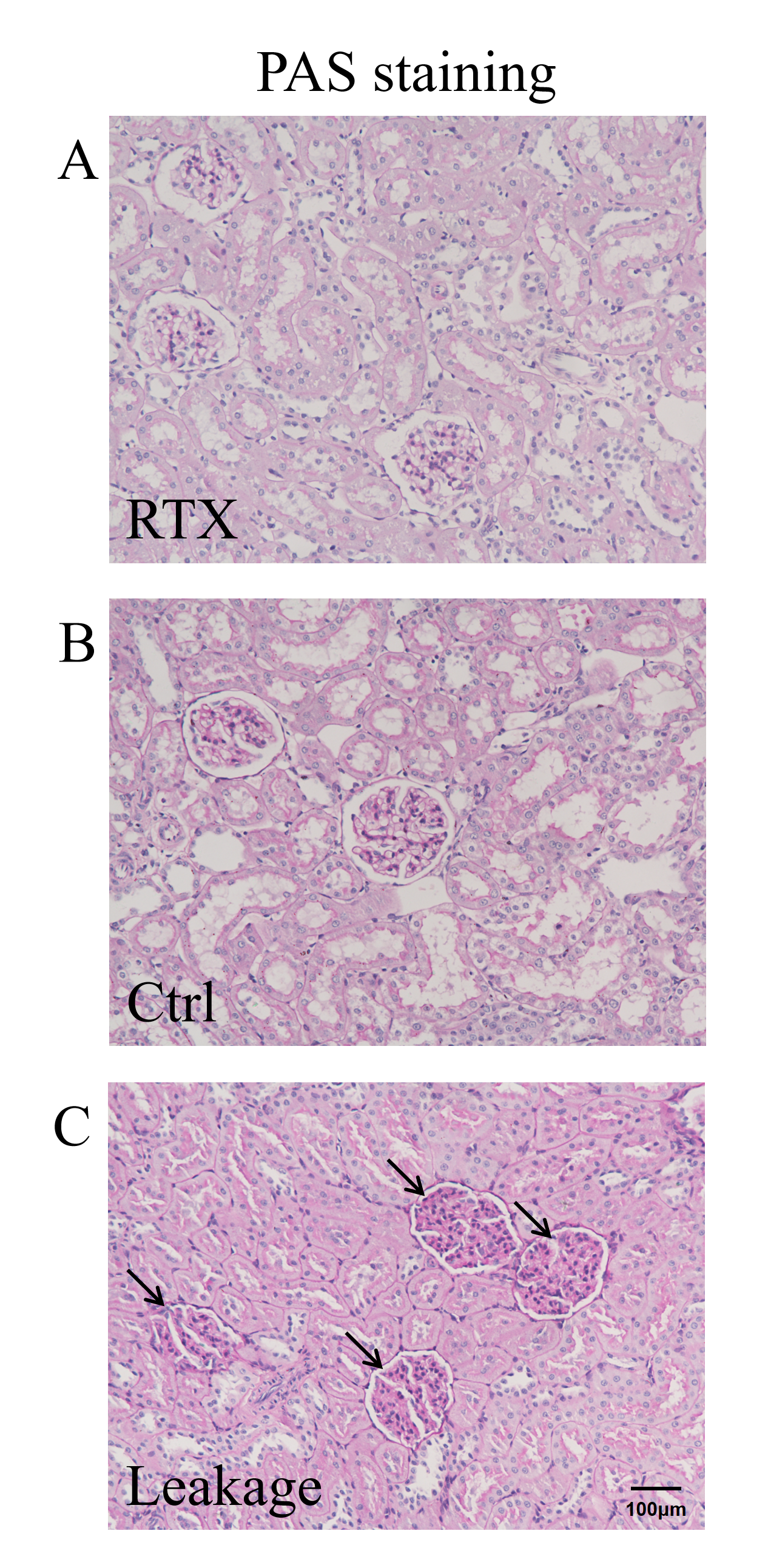

Supplement: FIGURE S4 — Effects of the deafferentation of PrAT by RTX on the kidneys. Injury occurred within glomeruli (glomerulosclerosis marked by arrows) with the leakage of RTX (C). In our study, RTX treatment (A) did not result in pathological changes in the kidneys (B). [file Image_4.TIF]

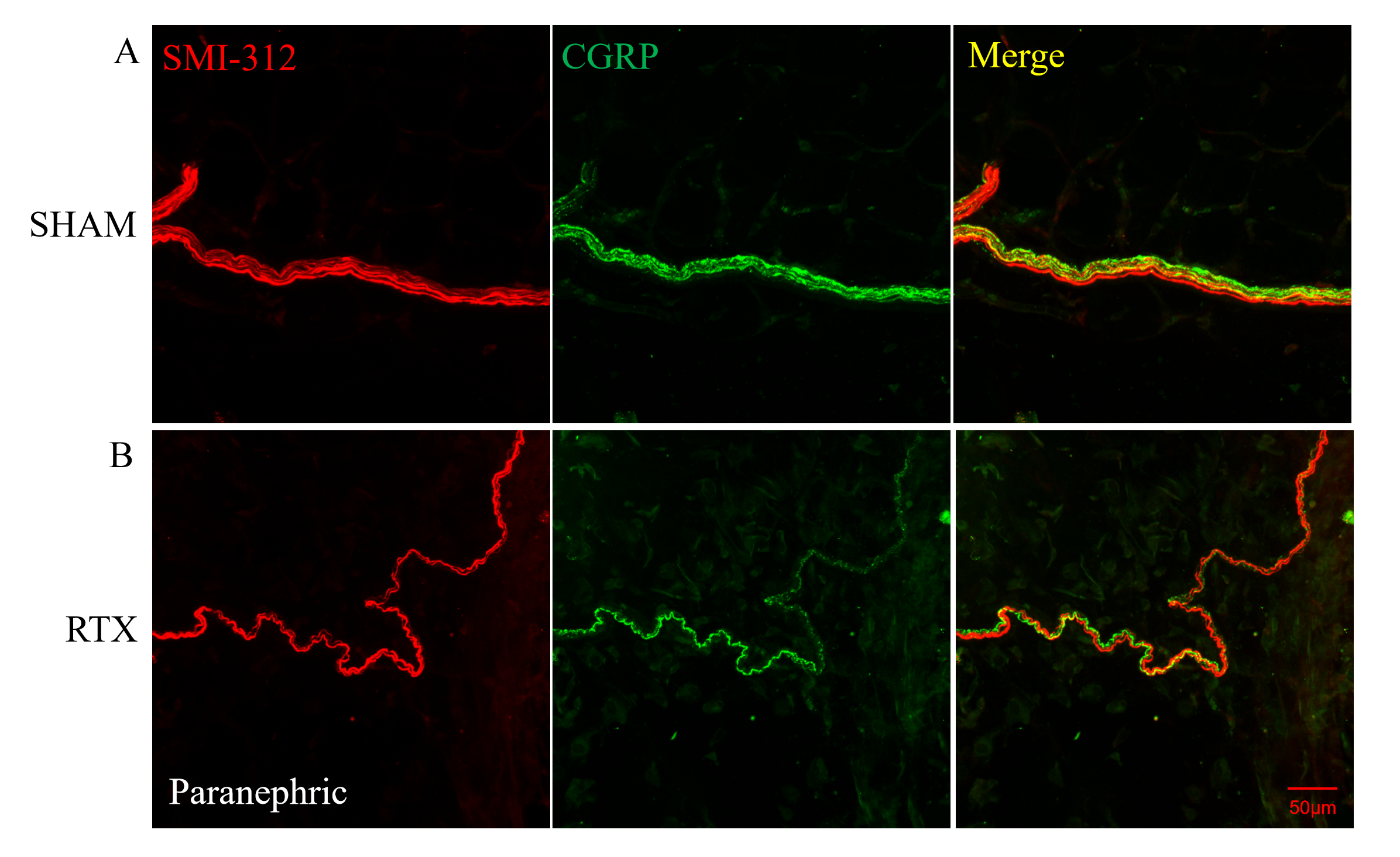

Supplement: FIGURE S5 — The deafferentation of PrAT using RTX (B) did not alter the density of sensory nerve fibers within paranephric adipose tissue compared to the control group (A). [file Image_5.tif]
